# Soil bioremediation approaches for petroleum hydrocarbon polluted environments

**DOI:** 10.3934/microbiol.2017.1.25

**Published:** 2017-01-19

**Authors:** Eman Koshlaf, Andrew S Ball

**Affiliations:** 1 Centre for Environmental Sustainability and Remediation, School of Science, RMIT University, Bundoora, Victoria 3083, Australia; 2 Department of Biology, Faculty of Science Algabal Algarbi University, Gharian, Libya

**Keywords:** petroleum hydrocarbon, bioremediation, contamination, hydrocarbon degrading microbes, environment

## Abstract

Increasing industrialisation, continued population growth and heavy demand and reliance on petrochemical products have led to unprecedented economic growth and development. However, inevitably this dependence on fossil fuels has resulted in serious environmental issues over recent decades. The eco-toxicity and the potential health implications that petroleum hydrocarbons pose for both environmental and human health have led to increased interest in developing environmental biotechnology-based methodologies to detoxify environments impacted by petrogenic compounds. Different approaches have been applied for remediating polluted sites with petroleum derivatives. Bioremediation represents an environmentally sustainable and economical emerging technology for maximizing the metabolism of organic pollutants and minimizing the ecological effects of oil spills. Bioremediation relies on microbial metabolic activities in the presence of optimal ecological factors and necessary nutrients to transform organic pollutants such as petrogenic hydrocarbons. Although, biodegradation often takes longer than traditional remediation methods, the complete degradation of the contaminant is often accomplished. Hydrocarbon biodegradation in soil is determined by a number of environmental and biological factors varying from site to site such as the pH of the soil, temperature, oxygen availability and nutrient content, the growth and survival of hydrocarbon-degrading microbes and bioavailability of pollutants to microbial attack. In this review we have attempted to broaden the perspectives of scientists working in bioremediation. We focus on the most common bioremediation technologies currently used for soil remediation and the mechanisms underlying the degradation of petrogenic hydrocarbons by microorganisms.

## 1. Introduction

Increasing industrialisation, continued population growth and heavy demand and reliance on petrochemical products have led to unprecedented economic growth and development. However, inevitably this dependence on fossil fuels has resulted in serious environmental issues over recent decades. Currently, petroleum production represents a major cause of ecosystem problems. World annual petroleum production is predicted to reach twelve million metric tonnes. British Petroleum [1] report that globally, oil production and consumption grew by 2.1 million barrels per day (b/d) (∼2.3%) in 2014. However it has been estimated that between 1.7 to 8.8 million metric tonnes of oil from natural and anthropogenic sources are released into the environment annually [2].

Due to this, the effect of petroleum hydrocarbons on the ecosystem, including their eco-toxicity and the potential implications they pose for both environmental and human health is a current area of research focus. In particular soil pollution has been and remains a severe and widespread environmental hazard attracting considerable public and scientific attention. Much of this pollution has resulted from the increased activities associated with petroleum exploration, transport and processing. In addition, the lack of waste oil recycling and the disposal of hazardous oil wastes into landfills without sufficient management has further increased the number of contaminated sites. For instance, during 2005 almost nine oil pollution incidents are reported around the world every day, in addition to an estimation of a yearly oil spill of one million tonnes into the UK terrestrial environment alone [3]. In the USA around 90% of the contaminated sites are petroleum hydrocarbon contaminated soils [3],[4]. Crude oil is a complex mixture of aliphatic and aromatic hydrocarbon, compounds that are frequently reported as soil pollutants [5]. Due to the mobility of petrogenic hydrocarbons together with their toxicity, mutagenicity and carcinogenicity, soil contamination is considered as a major challenge for healthy environments [6]. The carcinogenic effects of particular petroleum hydrocarbons is well established with an observed increase in cancer incidences in petroleum-associated workers including skin, lung, bladder, liver and stomach cancers in addition to reproductive, neurologic and developmental effects [7],[8].

There is a clear and urgent need to remediate petroleum hydrocarbon-contaminated areas around the world and several traditional physio-chemical methods such as soil washing, soil vapour extraction, incineration, the use of oil booms and solidification are available for oil spills remediation. Table 1 summarises the benefits and limitations of these approaches. However, many of these approaches are disruptive, labour intensive and relatively expensive processes requiring plenty of time and resources [9]. In the USA for example, the costs of soil contaminant removal was estimated to be more than 1 trillion US dollars [3]. Furthermore, the basic costs for removal of pollutants from large-scale commercial sites costs as a minimum of $US200,000 with an additional $US 40–70 for each cubic metre of contaminated soil [10].

Since 2000, remediation strategies based on microbial degradation capabilities (bioremediation) have received extensive attention and have become a current research focus especially in terrestrial environments. Bioremediation is the use of microorganisms, mainly bacteria and fungi, or plants to utilise and break down environmental contaminants such as petroleum into less harmful substances. These techniques have a number of key advantages over traditional technologies including the fact that they are simple to implement, environmentally friendly, applicable over large areas, cost-effective and can lead to the complete destruction of different contaminants [11]. However like all technologies there are some limitations associated with this technology. These include the extended treatment time, low predictability and dependence on environmental factors.

**Table 1.** Summary of bioremediation techniques for hydrocarbon contaminated soils.

Remediation strategy	Example of method	Treating site	Cost (US $/m^3^) ^a^	Benefits	Limitations
**Physical**	Vapour extraction	*Ex situ*	405–1,485	Fast Permanentremoval of pollutants Ideal for high levels of pollution	Costly Destructive Prone to secondary pollution
**Chemical**	Thermal desorption	*Ex situ*	80–440	Fast Dose not generatelarge volumes of waste material Ideal for high level of contamination	Costly Destructive Prone to secondary pollution
**Biological**	Biostimulation	*In situ*	30–100	Environmentally friendly Cost effective Minimum site disruption Useful for low level of pollutants	Require longer time Low predictability Reliant on environmental factors

## 2. Chemical Composition of Petroleum Hydrocarbons

**Figure 1.**
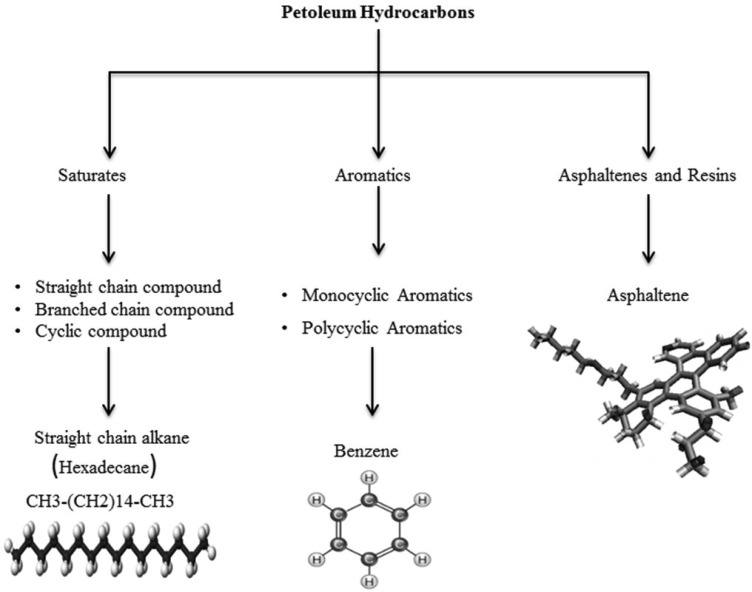
The various fractions of hydrocarbons that comprise crude oil.

From a chemical point of view, the term crude oil is strictly ascribed to a complex mixture of organic compounds comprising predominantly hydrogen and carbon atoms, but also containing smaller quantities of nitrogen, oxygen, sulphur along with traces of metallic constituents [12],[13]. High-resolution Gas Chromatography (GC) equipped with flame-ionization detection (FID) and capillary GC-Mass Spectrometry (MS) are the most important and most commonly employed techniques for oil compounds separation, characterization and identification [14]. More than seventeen thousand distinct chemical compounds in crude oil have been identified, making it perhaps the most complicated natural mixture of organic components [15]. Petrogenic hydrocarbons can be divided into four fractions: the aliphatic fraction (saturates), the aromatic fraction, the asphaltene fraction (phenols, fatty acids, ketones, esters and porphyrins), and the resins (pyridines, quinolines, carbazoles, sulfoxides, and amides) (Figure 1) [15]. A description of each of these groups follows.

### 2.1. Aliphatic hydrocarbons (saturates)

Aliphatic hydrocarbons represent the major component of crude oil and petroleum products Petroleum contamination in the US is dominated by diesel in which aliphatic hydrocarbons represent up to 90% by volume of the petroleum products [3]. According to their chemical structure, saturates are classified into groups including alkanes (paraffins) and cycloalkanes (naphthenes). Unlike aromatic hydrocarbons, aliphatic hydrocarbons are methane derivatives, which are both non-aromatic and non-cyclic organic compounds, containing both saturated and unsaturated linear or branched open-chain structures [3]. In an oil spill short chain aliphatic alkanes generally volatilise rapidly from the parent oil. However these compounds may also spread over solid and water surfaces, entering muddy or sandy sediments where they may remain exerting a toxic impact on the ecosystem [16],[17]. Aliphatic hydrocarbons with larger chain lengths (C20–C40) are more persistent in the soil, not readily volatilised and difficult to degrade because of their low water solubility, biological availability and structure [3].

### 2.2. Aromatic hydrocarbons

**Figure 2.**
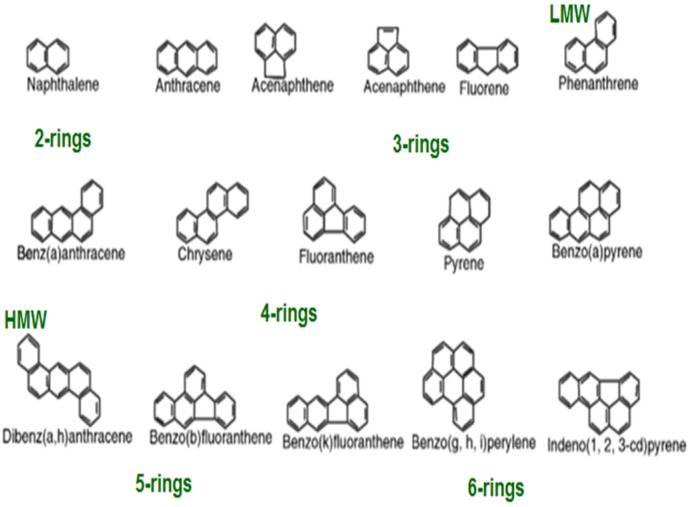
Structures and nomenclatures of the 16 PAHs on the US EPA priority pollutant list.

Aromatic fractions of organic compounds are classified by the presence of two to six aromatic rings (e.g. fused benzene ring) with or without alkyl substituents arranged in linear, cluster or angular configurations within their structure [18]. Polynuclear aromatic hydrocarbons (PAH) are of major concern to both public and environmental health due to their acute toxicity, as well as their mutagenic and carcinogenic properties [19]. These compounds are end products of crude oil processing; they comprise on average approximately 26–30% of oil constituents [19]. PAHs are the major components of creosote, (a complex mixture of about 200 compounds, including phenolic and heterocyclic contaminants) [19]. Physical-chemical characteristics of such compounds differ with their molecular weight and number of benzene rings. The distribution of PAHs through air, soil, and water and their fate, transport and impacts on the ecosystem are dependent on their physicochemical properties. Those compounds with a high molecular weight have low, chemical reactivity, solubility in water and volatility; however, they are highly carcinogenic [20]. PAHs display a number of common features including sensitivity to light, and heat and corrosion resistance [21]. PAHs, especially those with increased complexity and molecular weight are toxic and persistent compounds. They are generally considered to be of long term environmental significance. PAHs are relatively recalcitrant in soil, having high hydrophobicity making them increasingly difficult to be degraded [14],[22]. The U.S. Environmental Protection Agency (US-EPA) has listed sixteen PAH compounds as priority pollutants (Figure 2). Some of these compounds are considered to be carcinogenic; hence significant attention has been paid to limit their distribution in the environment and the degradation of these pollutants.

### 2.3. Asphaltenes and resins

**Figure 3.**
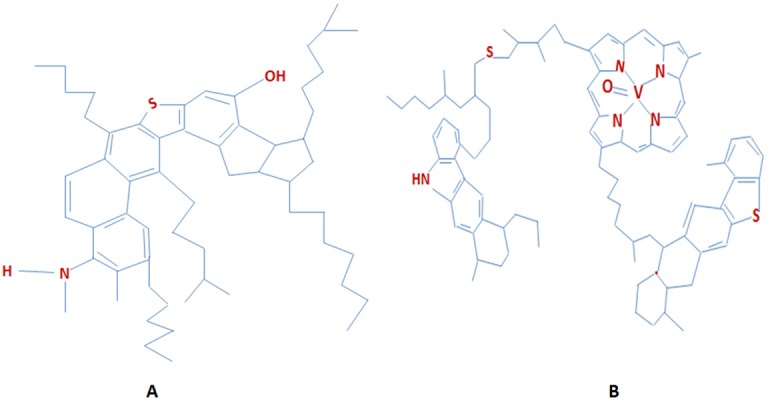
Typical molecular structures of (a) resins and (b) asphaltenes.

The properties of asphaltenes (phenols, fatty acids, ketones, esters and porphyrins) and resins (pyridines, quinolines, carbazoles, sulfoxides, and amides) have an impact on the behaviour of crude oil during production and refining. They constitute almost 10% of crude oil composition [23],[24]. Asphaltenes and resins have complex structures consisting of more polar compounds. They contain nitrogen, oxygen and sulphur along with carbon (Figure 3) [25],[26]. Asphaltenes are not crystallized and are unstable compounds which form a separate non aqueous layer [24]. Due to their structural similarity and composition (generally polar, polynuclear molecules composed of aromatic rings, aliphatic side chains and a few heteroatoms), resins has been considered to display a strong affinity to asphaltenes [26]. Resins and asphaltenes are generally considered as being resistant to microbial attack.

## 3. Hazards of Petroleum Hydrocarbon Contamination

Once petroleum hydrocarbons reach an environment, primary biological damage occurs by blocking the supply of water, nutrients, oxygen and light, affecting soil fertility, plant growth and germination [12],[27]. PAHs in the environment are mainly found in soil and sediment at various concentrations causing significant environmental damage [28]. After they mix with water, PAHs tend to seep into the ground, where they persist, reducing the quality and productivity of the soil making it unsuitable for cultivation and investment [29]. PAHs are poisonous at low concentrations and they can be carcinogenic or mutagenic to wildlife and humans. Uptake of such recalcitrant chemicals from contaminated soil may occur through ingestion, inhalation or dermal exposure to contaminated soil or dust. Since petrogenic hydrocarbons persist in the ecosystem for long periods of time, they can accumulate in animals and plant tissue, passing from one to the next through the food chain causing death or genetic mutations in animals and humans [30]. Frequent exposure to sub-lethal doses of these compounds can cause several physiological impairments, leading to a number of health impacts including liver damage, haemolytic anaemia, weight loss, gastrointestinal deterioration, impaired immune system and reduced productivity [31]. The presence of aliphatic hydrocarbons can also adversely affect soil microflora and structure producing oil films and slicks and limiting the interchange of oxygen and nutrient in the soil [32],[33]. Aliphatic hydrocarbons may also affect the nervous system, causing dizziness, headaches, fatigue and limb numbness, tremors, temporary limb paralysis and unconsciousness at high concentrations [34].

## 4. Fate of Petroleum Hydrocarbon in the Environment

**Figure 4.**
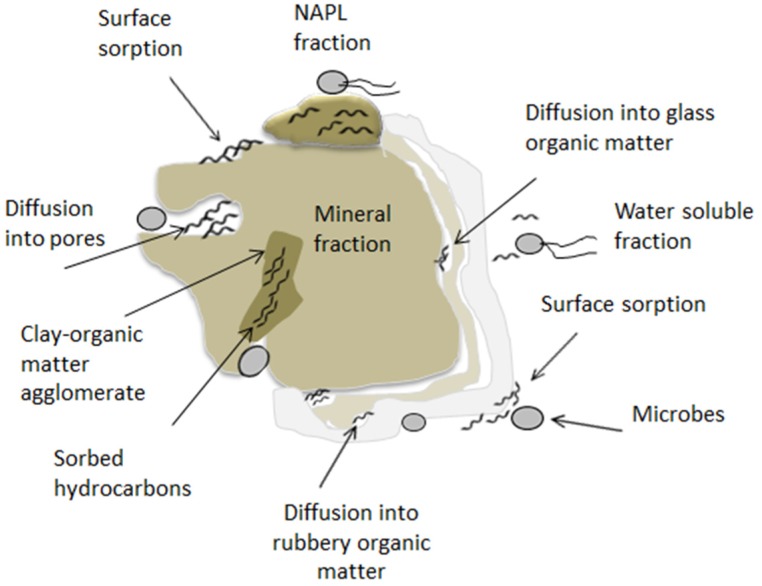
Possible interactions between soil matrices and hydrocarbons redrawn from [3].

Considering the nature and extent of hydrocarbon pollution in soils and in order to predict how successful oil remediation approaches will be, understanding the fate and behaviour of such contaminants in the environment is vital. During an oil spill, weathering occurs and the oil is subjected to a variety of physicochemical processes [35]. These processes can alter the composition and properties of the oil affecting the degree of hydrocarbon degradation, sequestration and interaction with soil microbes. The fate and spread of these compounds on the subsurface dependson the viscosity and quantity of the oil. In the terrestrial environment, the fate of petroleum hydrocarbons is influenced by (a) the composition and physical properties of the soil such as particle size, porosity, organic matter content, permeability and surface area and (b), the physical and chemical properties and composition of petroleum products including air diffusion coefficient, solubility in water and boiling point [36]. The biodegradability of petroleum hydrocarbons can also be affected by the concentration and bioavailability of the contaminants. Hydrocarbon bioavailability refers to the fraction of contaminants that can be utilized or transformed by the soil microbial community. Sorption is also an important factor influencing the complete degradation of organic pollutants in the soil. Reduced sorption of the hydrocarbon fraction increases resistance to desorption resulting in increased persistence within the soil organic matrix. In contaminated soil, two hydrocarbon fractions should be considered when choosing bioremediation treatment: firstly the irreversibly adsorbed hydrocarbons; this fraction is not bioavailable and considered to be non-biodegradable. The second portion is the bioavailable fraction which is able to desorb and diffuse in the solid particles as a water soluble fraction [37]. Petroleum hydrocarbons can be fractionated and sequestered within the soil via sorption to organic matter or diffuse into the three-dimensional structure of the organic matter. Figure 4 summarizes these interactions [3], [38]. Following the initial oil spill the physical interactions become more complicated; this known as aged contamination [38].

The biodegradable fraction of organic pollutants in soils is the fraction that is easily desorbed to or from the soil particles and exist in the aqueous phase. It is well established that as the interaction between soil particles and pollutants increase, there will be a proportional reduction in contaminant extraction and biodegradation [39]. Hydrocarbon fractions that are more tightly sorbed onto soil organic matter are more recalcitrant and resistant to degradation compared to volatile or soluble hydrocarbons. This is a very important consideration when designing or applying a strategy for the degradation of contaminated soils as petrogenic hydrocarbons tend to strongly adsorb to the soil [40].

## 5. Microbial Degradation of Petrogenic Hydrocarbons

Microorganisms have the ability to metabolize many organic contaminants, using them as an energy source or converting them to non-toxic products (carbon dioxide, water and biomass). Different microbial electron acceptors such as oxygen, nitrate, manganese, iron and sulphate can be involved in the biotransformation of aliphatic and aromatic hydrocarbons. Microorganisms can activate and oxidize hydrocarbon compounds. The addition of one or two hydroxyl groups to the hydrocarbon skeleton represents the first step in the aerobic catabolism of hydrocarbons. During hydrocarbon degradation, activation is achieved through different enzymes, for example by the introduction of molecular oxygen to the substrate (catalysed by oxygenases), the addition of two hydroxyl groups (catalysed by dioxygenases) or the addition of one atom of oxygen into the hydrocarbon (catalysed by monooxygenases) [41]. Activation is accomplished through two different mechanisms; aerobically, oxygen is used as an electronic accepter and anaerobically catabolism occurs at slower rates compared with aerobic microbial degradation [42].

### 5.1. Microbial degradation of aliphatic compounds

**Figure 5.**
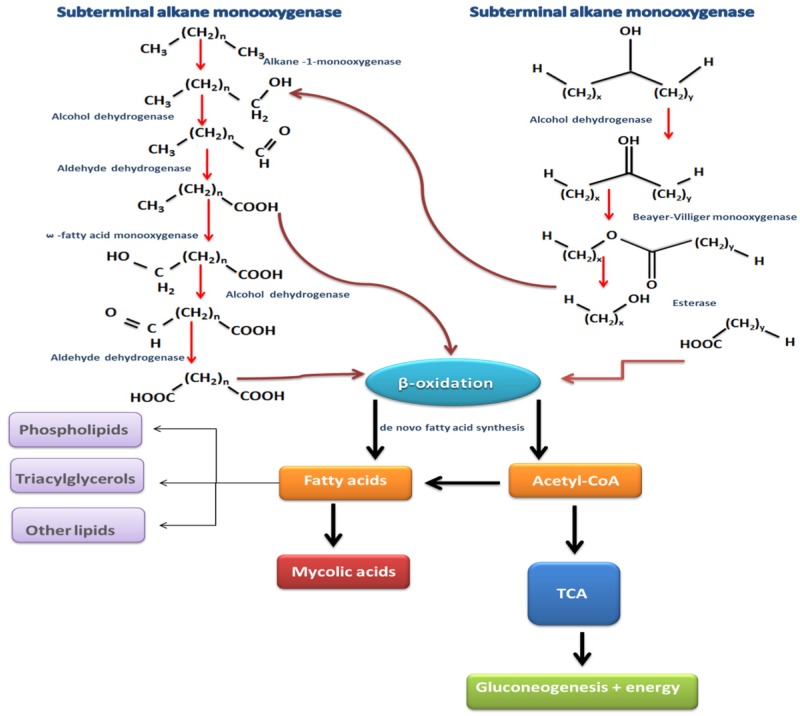
The main n-alkanes degradation pathways (terminal and subterminal oxidation). Redrawn from [48].

**Figure 6.**
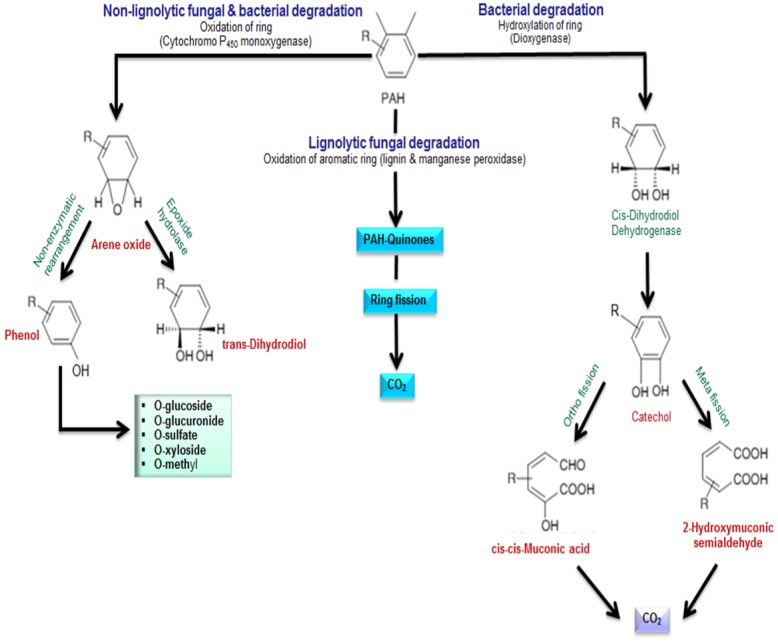
Aromatic hydrocarbon breakdown pathways in bacteria and fungi. Redrawn from [18],[47].

Microbial degradation of petroleum hydrocarbon compounds is carried out by a range of microbial groups capable of degrading a wide range of target constituents present in oil contaminated environments. A biodegradation pathway is a gradual transformation of organic contaminants into intermediates of the central intermediary metabolism. For example, in the case of aliphatic hydrocarbons (n- alkanes), microorganisms utilise soluble or integral-membrane non-haem iron monooxygenases; these enzymes, alkane hydroxylases (e.g. AlkB) hydroxylate the substrate [41]. Essentially, the aerobic degradation of alkanes is usually initiated with an oxidization of the terminal methyl group producing a primary alcohol. This product is further oxidised by alcohol and aldehyde dehydrogenases to form the corresponding aldehyde. The resulting product is finally converted to fatty acid via oxidation. Fatty acid couples with CoA and is then channeled into the β-oxidation pathway in the form of acetyl-CoA (Figure 5). Long-chain alkanes are degraded via terminal as well as sub-terminal oxidation [43]. In the case of sub-terminal oxidation the generated secondary alcohols are transformed to the corresponding ketone and this is converted to an ester through an oxidation step via a Baeyer-Villiger monooxygenase, and then hydrolysed with an esterase to generate an alcohol and a fatty acid (Figure 6).

### 5.2. Microbial degradation of aromatic compounds

Hydrocarbon-degrading microorganisms such as *Pseudomonas*, *Rhodococcus*, *Sphingobium* and *Sphingomonas* spp. are ubiquitous in the ecosystem. These microbes are capable of degrading aromatic hydrocarbons; the catabolism process commences with an oxidation step of one of the aromatic rings and the structured fracture of the substrate to PAH metabolites and CO_2_. Fundamentally, the reaction is catalyzed by aromatic hydrocarbon ring hydroxylating enzymes (ARHDs) and form *cis*-dihydrodiols. The dihydrodiols are then oxidized via a dehydrogenase reaction to produce PAH dihydroxy derivatives which are further exposed to the action of ring cleaving dioxygenases. The action of aromatic ring cleavage is accompanied by integration of a pair of oxygen atoms into the dihydroxy derivative molecule. Dihydroxylated intermediates may then be cleaved through either an intradiol manner (*ortho*) cleavage pathway, or through the intradiol ring (*meta*) cleavage pathway, with the formation of catechols and subsequently metabolised to carbon dioxide and water through the TCA (tricarboxylic acid cycle) cycle [40] (Figure 6). A small number of bacteria oxidise PAHs via the cytochrome P450 monooxygenase, resulting in the production of trans-dihydrodiols [44]. There is only broad specificity between PAH degradation and the catalyzed enzymes (ARHDs). Some key factors play a fundamental role in the specificity of PAH transformation by bacteria. In particular the activity of an ARHD is dependent on the specific PAH. The bacterial metabolic pathways for PAH degradation are well documented in the literature [45],[46]. Additionally, several fungi, capable of metabolizing PAH contaminants have been identified. During the fungal mineralization of PAH two different metabolic pathways are involved. Non-ligninolytic fungi deal with PAHs using the cytochrome P450 monooxygenase pathway, where PAHs are oxidised to arene oxides (the primary products of PAH metabolism) via the incorporation of a single oxygen atom into the ring of the substrate [47]. In contrast, white-rot fungi (a ligninolytic fungus), mineralize PAHs using a soluble extracellular ligninolytic enzyme such as manganese peroxidase, laccases and lignin peroxidase (Figure 6).

Since lignin contains a selection of aromatic compounds, these enzymes participate in the oxidation of lignin as well as different organic complexes [49]. Some fungi can produce more than one enzyme: for example, non-ligninolytic and ligninolytic enzymes can be produced by numerous ligninolytic fungi (e.g. *Pleurotus ostreatus* and *Phanerochaete chrysosporium*) [47]. Principally, the aerobic degradation of aromatic hydrocarbons by bacteria and fungi is accomplished through three different pathways (Figure 6).

## 6. Petroleum-utilizing Microorganisms (Abundance and Diversity)

Hydrocarbonoclastic microorganisms are the main agents of the degradation of petroleum hydrocarbons, owing to their associated metabolic capabilities (Table 2). Reviews on the degradation of petrogenic hydrocarbons have confirmed that numerous microbes (mainly bacteria and fungi) are capable of the degradation of petroleum hydrocarbons, utilising them as the sole carbon source for metabolism and energy. Bacteria are the most active petroleum degrading agents; they work as primary degraders of a wide range of target constituents present in soil, water and sludge [50]. Organisms belonging to various genera have been reported as hydrocarbonoclastic exhibiting the potential for the degradation of different fractions of petrogenic hydrocarbons; many of these organisms have been isolated from either soil or aquifers (Table 2). Typical bacterial groups include *Mycobacterium* spp., *Arthrobacter* spp., *Marinobacter* spp., *Achromobacter* spp., *Alcaligenes* spp., *Corynebacterium* spp*., Flavobacter* spp*., Micrococcus* spp., *Nocardia* spp. and *Pseudomonas* spp., [51]. More recently, scientists have reported the isolation of other bacterial genera capable of oxidising and degrading a wide range of hydrocarbons of crude oil (n-alkanes and aromatic hydrocarbons). Among these organisms are the genera *Bacillus*, *Dietzia, Gordonia, Halomonas, Cellulomonas, Rhodococcus* and halotolerant *Alcanivorax* spp. [52]–[55]. Several studies have also reported that a diverse group of fungi, such as those belonging to the genera *Aspergillus* spp., *Penicillium* spp., *Cunninghamella* spp., *Fusarium* spp., *Saccharomyces* spp., *Amorphoteca* spp., *Syncephalastrum* spp., *Neosartorya* spp., *Phanerochaete* spp., *Paecilomyces* spp., *Talaromyces* spp. *and Graphium* spp. are capable of mineralizing petroleum hydrocarbons with varying degradation rates [51],[56]. Numerous filamentous fungi as well as white-rot fungi have also shown the capability to oxidise and dissipate a wide range of PAHs into several harmless metabolic products. For instance, *Cunninghamella elegans*, a filamentous non-ligninolytic fungus was isolated from soil and has been implicated in the transformation and degradation of several PAH compounds including benzo[a]pyrene, 9,10-dihydrobenzo[a]pyrene and benz[a]anthracene [57]. *Psilocybe* spp., *Cyclothyrium* spp. and *Penicillium simplicissimum* are additional examples of filamentous fungi which have been reported to exhibit hydrocarbonoclastic activities against different PAHs [49].

**Table 2.** Isolated bacterial strains reported to exhibit hydrocarbonoclastic activity. Recreated from [42],[46],[58].

Species/Strain	Substrate	Species/Strain	Substrate
***Achromobacter sp.* NCW**	CBZ	*Geobacillus thermodenitrificans* NG80-2	C_15_–C_36_
***Acinetobacter baylyi* ADP1**	−C_36_	*Gordonia sp.* TY-5	C_3_, C_13_–C_22_
***A. calcoaceticus* 69-V**	C_11_–C_18_	*Janibacter sp.* YY-1	DBF, FLE, DBT, PHE, ANT, DD
***A. calcoaceticus* EB104**	C_6_–C_18_	*Marinobacter* NCE312	NAP
***A. calcoaceticus* NCIB 8250**	C_8_–C_16_	*Marinobacter sp.* BC36, BC38, & BC42	C_18_
***Acinetobacter sp.* 2796A**	C_10–_C_16_	*Marinobacter hydrocarbonoclasticus* 617	C_16_–C_30_
***Acinetobacter sp.* M-1**	C_13_–C_44_	*Mycobacterium avium*	paraffins
***Acinetobacter calcoaceticus* RR8**	C_10_–C_34_	*M. avium subsp. paratuberculosis*	paraffins
***Acinetobacter lwoffi***	C_12_–C_28_	*M. bovis* BCG	C_12_–C_16_
***Acinetobacter sp*. ODDK71**	C_12_–C_30_	*M. smegmatis*	C_9_–C_16_
***Acinetobacter sp*. S30**	−C_33_	*M. tuberculosis* H37Rv	C_11_–C_16_
***Acinetobacter sp.* DSM17874**	C_10_–C_40_	*Mycobacterium sp.* CH1	C_12_–C_28_
***Alcanivorax borkumensis* AP1**	C_10_–C_20_	*Mycobacterium sp.* HXN 600	C_6_–C_24_
***Alcanivorax borkumensis* SK2**	C_8_–C_32_	*Mycobacterium sp.* OFS	C_11_–C_28_
***Alcaligenes odorans* P20**	−C_33_	*Mycobacterium sp.*	PYR, BaP
***Alcaligenes denitrificans***	FLA	*Mycobacterium sp*.JS14	FLA
***Arthrobacter nicotianae* KCC B35**	C_10_–C_40_	*Mycobacterium sp*. 6PY1, KR2, AP1	PYR
***Arthrobacter sp*.F101**	FLE	*Mycobacterium sp.* RJGII-135	PYR,BaA, BaP
***Arthrobacter sp.* P1-1**	DBT, CBZ, PHE	*Mycobacterium sp.* PYR-1, LB501T	FLA, PYR, PHE, ANT
***Arthrobacter sulphureus* RKJ4**	PHE	*Mycobacterium sp*. CH1, BG1, BB1, KR20	PHE, FLE, FLA, PYR
***Acidovorax delafieldii* P4-1**	PHE	*Mycobacterium flavescens*	PYR, FLA
***Bacillus cereus* P21**	PYR	*Mycobacterium vanbaalenii* PYR-1	PHE, PYR, dMBaA
***Bacillus thermoleovorans* B23 & H41**	C_9_–C_30_	*Mycobacterium sp. KMS*	PYR
***Bacillus thuringiensis/cereus* A2**	C_6_–C_28_	*Nocardioides aromaticivorans* IC177	CBZ
***Brevibacteriumsp*. HL4**	PHE	*Nocardioides sp.* CF8	C_2_–C_16_
***Burkholderia sp*.S3702, RP007, 2A 12TNFYE-5, BS3770**	PHE	*Paracoccus sereniphilus/marcusii* A7	C_6_–C_28_
***Burkholderia cepacia* ATCC 25416**	C_10_–C_16_	*Paracoccus sp. strains* Ophe1 & Sphe1	C_10_–C_28_
***Burkholderia cepacia* RR10**	C_12_–C_34_	*Pasteurella sp*. IFA	FLA
***Burkholderia sp*.C3**	PHE	*Planococcus alkanoclasticus* MAE2	C_11_–C_33_
***Burkholderia cepacia* BU-3**	NAP, PHE, PYR	*Polaromonas naphthalenivorans* CJ2	NAP
***Burkholderia cocovenenans***	PHE	*Prauserella rugosa* NRRL B-2295	C_8_–C_14_
***Burkholderia xenovorans* LB400**	BZ, BP	*Pseudomonas aureofaciens* RWTH 529	C_10_
***B. mallei***	C_10_–C_16_	*Pseudomonas sp.* 7/156	n. d
***B. pseudomallei***	C_10_–C_16_	*Pseudomonas putida* GPo1	C_5_–C_12_
***Chryseobacterium sp*. NCY**	CBZ	*P. putida* P1	C_8_
***Cycloclasticus sp.*P1**	PYR	*Pseudomonas fluorescens* CHA0	C_12_–C_32_
***Brachybacterium sp.***	C_10_–C_20_	*Pseudomonas aeruginosa* PAO1	C_12_–C_24_
***Desulfatibacillum aliphaticivorans* CV2803**	C_13_–C_18_	*P. aeruginosa* PG201	C_10_–C_16_
***Dietzia cinnamea* P4**	C_11_–C_24_	*P. aeruginosa* KSLA473	C_5_–C_16_
***Dietzia psychralcaliphila***	C_13_–C_24_	*P. aeruginosa* NCIMB 8704 & 9571	C_8_–C_16_
***P. aeruginosa* ATCC 17423**	C_8_–C_16_	*R. erythropolis* Q15	C_8_–C_32_
***P. aeruginosa* RR1**	C_12_–C_34_	*R. erythropolis* 35-O	C_6_–C_16_
***P. aeruginosa strains* A1, A3, A4, A5, A6**	C_6_–C_28_	*R. erythropolis* 23-D	C_6_–C_36_
***Pseudomonas sp.* PUP6**	C_12_–C_28_	*R. erythropolis* NRRL B-16531	C_6_–C_36_
***Pseudomonas sp*.C18, PP2, DLC-P11**	NAP, PHE	*R. erythropolis* 42-O	C_6_–C_32_
***Pseudomonas sp*.BT1d**	HFBT	*R. erythropolis* 62-O	C_6_–C_16_
***Pseudomonas sp*.B4**	BP, CBP	*R. erythropolis* 23-D	C_6_–C_36_
***Pseudomonas sp*.HH69**	DBF	*R. erythropolis* 50-V	C_6_–C_32_
***Pseudomonas sp.*CA10**	CBZ, CDD	*R. erythropolis* NRRL B-16531	C_6_–C_36_
***Pseudomonas sp.* NCIB 9816-4**	FLE, DBF, DBT	*Rhodococcus fascians* 115-H	C_6_–C_32_
***Pseudomonas sp*. F274**	FLE	*R. fascians* 154-S	C_6_–C_24_
***Pseudomonas paucimobilis***	PHE	*Rhodococcus rhodochrous*	C_12_–C_20_
***Pseudomonas vesicularis* OUS82**	FLE	*Staphylococcus sp*. PN/Y	PHE
***Pseudomonas putida* P16, BS3701, BS3750, BS590-P, BS202-P1**	NAP, PHE	*Stenotrophomonas maltophilia* VUN 10,010	PYR, FLA, BaP
***Pseudomonas putida* CSV86**	MNAP	*S. maltophilia* VUN 10,003	PYR, FLA, BaA, BaP, DBA, COR
***Pseudomonas fluorescens* BS3760**	PHE, CHR, BaA	*Sphingomonas yanoikuyae* R1	PYR
***Pseudomonas stutzeri* P15**	PYR	*Sphingomonas yanoikuyae* JAR02	BaP
***Pseudomonas saccharophilia***	PYR	*Sphingomonas sp*.P2, LB126	FLE, PHE, FLA, ANT
***Pseudomonas aeruginosa***	PHE	*Sphingomonas sp.*	DBF, DBT, CBZ
***Ralstonia sp*. SBUG 290 U2**	DBF NAP	*Sphingomonas paucimobilis* EPA505	FLA, NAP, ANT, PHE
***Rhodanobacter sp.* BPC-1**	BaP	*Sphingomonas wittichii* RW1	CDD
***Rhodococcus sp.***	PYR, FLA	*strain* AK01	C_13_–C_18_
***Rhodococcus sp*. 1BN**	C_6_–C_28_	*strain* HdN1	C_14_–C_20_
***Rhodococcus sp*. RR12 & RR14**	C_14_–C_34_	*strain* Hxd3	C_12_–C_20_
***Rhodococcus sp. strains* T12 & TMP2**	C_9_–C_22_	*Terrabacter sp*.DBF63	DBF, CDBF, CDD, FLE
***Rhodococcus sp*. NCIM5126**	C_13_–C_20_	*Thalassolituus oleivorans*	C*_7_*–C_20_
***Rhodococcus sp*.WU-K2R**	NAT, BT	*Thermooleophilum album*	C_13_–C*_20_*
***Rhodococcus erythropolis* I-19**	ADBT	*Thermus sp.* C2	C_9_–C_39_
***R. erythropolis* D-1**	DBT	*Weeksella sp*. RR7	C_12_–C_34_
***P. aeruginosa* ATCC 17423**	C_8_–C_16_	*Xanthamonas sp.*	PYR, BaP, CBZ
***P. aeruginosa* RR1**	C_12_–C_34_	*Xylella fastidiosa* RR15	C_14_–C_34_

Pyrene (PYR), anthracene (ANT), fluorene (FLE), dibenz[a,h]anthracene (DBA), naphthalene (NAP), phenanthrene (PHE), benz[a]anthracene (BaA), dimethylbenz[a]anthracene(dMBaA), chlorinated dibenzothophene (CDBF), benzothiophene(BT), alkylated dibenzothiophene (ADBT), 3-hydroxy-2-formylbenzothiophene (HFBT), chrysene (CHR), dibenzo-p-dioxin (DD), biphenyl; CBP, fluoranthene (FLA), chlorinated dibenzo-p-dioxin (CDD), benzo[a]pyrene (BaP), coronene(COR), methyl naphthalene (MNAP), carbazole (CBZ),chlorobiphenyl (BP), naphthothiophene (NAT), dibenzofuran (DBF), benzoate (BZ).

## 7. Bioremediation

Since there are many soil dwelling microbes that are capable of breaking down the diverse fractions of hydrocarbons and which survive under different conditions, each site may necessitate a specified bioremediation treatment. The different generic methods and strategies of bioremediation technologies being applied currently are natural attenuation, bioaugmentation, biostimulation and phytoremediation. The techniques are summarized in Table 3 and discussed in further detail below.

**Table 3.** The main characteristics of bioremediation technologies for petroleum-polluted soils.

Bioremediation method	Main features	Advantages	limitations
**1. Natural attenuation**	Utilising indigenous microbial populations under natural conditions	Cost effective	Requires extensive long-range observation Not always effective
**2. Bioaugmentation**	Addition of efficient pollutant of hydrocarbon-degrading microbes	Using a high biomass of hydrocarbon-degrading microbes	Requires extensive long-term monitoring Not always effective Poor adaptation of hydrocarbonoclastic microorganisms to the contaminated site Introduced strains can be inhibited by co-pollutants or native microorganisms
**Isolated strain**	Catalyse the degradation of single molecules or simple mixtures		
**Microbial consortium**	Catalyse the degradation of complex pollutantmixtures		
**3. Biostimulation**	Management of environmental factors (addition of nutrient)	More efficient than natural attenuation	Not always effective Optimal C/N/P ratio and pollutant bioavailability have to be determined
**Fertilizers**	Restoration of nutrient balance, C/N/P ratio optimization		
**(Bio)surfactants**	Stimulation of contaminant bioavailability		
**4. Phytoremediation**	Application of plants and theirassociated microorganisms	Supports hydrocarbon-degradingmicrobes within plant root	Pollutants toxic to the plant

### 7.1. Natural attenuation

By definition, natural attenuation is the simplest bioremediation process by which the indigenous microbial population (bacteria and fungi) eliminate or detoxify petroleum and other hydrocarbon pollutants hazardous to human health and/or the environment into less toxic forms in order to attenuate the polluted site. During this process the indigenous microbes utilise hydrocarbons as the sole carbon source based on their natural, metabolic pathways. This technology requires simply monitoring the process. When site pollution occurs, indigenous hydrocarbon-degrading microorganisms will increase rapidly and adapt to the freshly added pollutants resulting in contaminant degradation; however microbial diversity may be reduced [59]. This natural remediation process occurs naturally in most contaminated sites, and can be applied in areas where other restoration mechanisms cannot be applied or in relatively low polluted sites [60]. Biodegradation research has shown natural attenuation to be effective in petroleum contaminated sites, estimating that almost 25% of soils polluted with petrogenic hydrocarbon have been treated through natural attenuation [61]. One comparative study showed that natural attenuation can be as or even more effective than biostimulation and bioaugmentation methods and that the naturally occurring hydrocarbon degraders associated with the oil itself are capable of utilising hydrocarbons without any enhancement [62],[63]. Natural attenuation however is often limited by nutrient availability. In addition, microbial communities with high degrading activity may not be available on the site or may not possess the necessary catabolic genes for complete degradation, thus developed remediation practices are essential in this instances.

### 7.2. Bioaugmentation

The capacity of the microbial community in the soil to metabolize petroleum pollutants is determined by their structure and diversity [64]. In soils with insufficient or non-detectable numbers of indigenous pollutant-degrading microorganisms, natural attenuation perhaps is unsuitable as a remediation method, thus another bioremediation technologies should be applied. One of the alternate *in situ* bioremediation methods is bioaugmentation. This application involves the addition of single strains or consortia of hydrocarbon-degrading microbes (bacteria or to a lesser extent fungi) with catalytic capabilities to remediate contaminated sites in order to accelerate the biodegradation of undesired organic compounds. The bioaugmented hydrocarbon utilizers are normally isolated from petroleum hydrocarbon polluted environments [65]. The rationale behind bioaugmentation is that the introduction of hydrocarbon degrading microorganisms into polluted soil improves the biodegradative capacity of the indigenous population. Researches have reported that the application of bioaugmentation to contaminated marine and terrestrial environments exhibited superior treatment efficiency [66]–[69]. However, it has also been reported that bioaugmentation did not result in a significant increase in bioremediation and in some cases the inoculated hydrocarbon degrading microbes failed to show any degradation activity [70],[71]. In addition, the effect of introducing exogenous microorganisms on the diversity and activity of the natural ecosystem remains to be fully investigated. For example one recent study has shown that the addition of exogenous microbes led to significant changes in the composition of the soil microbial community [72].

### 7.3. Biostimulation

A widely practiced bioremediation technology that exploits the capability of microbes to degrade and/or detoxify petroleum pollutants in the soil is termed biostimulation. This procedure results in the stimulation of the growth and activity of the indigenous microorganisms present in the contaminated site through the addition of nutrients in order to accelerate the rate of natural biodegradation [73]. There exists extensive literature that have reported that high concentrations of petroleum hydrocarbon, containing around 80% carbon can lead to a rapid reduction in the concentration of inorganic nutrients present in the soil (e.g. nitrogen and phosphorus) [74]. Nitrogen is an example of a nutrient that is found in terrestrial environments in many forms. It is an essential nutrient which supports soil microbial growth and activity, increasing the rate of microbial cell growth, reducing the lag phase of microbes, supporting a large microbial population and, hence, increasing the rate of hydrocarbon degradation [75]. Biostimulation often includes the addition of nutrients and electron acceptors (such as P, C, N, and O_2_) and represents an effective technology for restoring oil polluted and nutrient deficient sites [76],[77]. However care must be taken in the amount of nutrients added; for example the addition of excess quantities of nitrogen may result in inhibition of the soil microbial community [78]. The main advantage of biostimulation is that enhanced biodegradation takes place by the native microbial communities which have already acclimatized to their environment. The biostimulation of native microbial communities of petroleum-impacted soil can be achieved in several ways. A wide range of organic and inorganic agents including nutrients, surfactants, fresh and composted sewage sludge and manure have been found to be successful biostimulators for petroleum hydrocarbon degradation [79],[80],[81]. Various laboratory and field experiments based on the addition of inorganic and organic fertilizers to the contaminated environment have shown positive impacts on hydrocarbon degradation; however a range of outcomes have been reported. For instance, stimulating the soil with inorganic nitrogen-phosphorus-potassium fertilization (NPK) and commercial products EAP and Terramend have been shown to stimulate the biodegradative activity of the native soil microbes [82]. In another study, soil amendment with manure increased the degradation rate of petroleum hydrocarbon up to 56% compared to that in the unamended soil samples (natural attenuation) (15.6%) [83]. In contrast other research results indicated that biostimulation did not significantly contribute to the degradation of petrogenic hydrocarbons in soil. For example, amendments including NPK, a compost extract and a microbial enrichment culture showed no significant impact on the remediation of diesel oil; in addition, no change in TPH concentration was observed when the soil was treated with coffee grains or horticultural waste. In this instance the authors concluded that the hydrocarbonclastic microbes preferred to consume the readily available carbon source (amendments) instead of petroleum hydrocarbons [84],[85]. Thus, it can be more valuable to characterise the polluted location, ecological conditions and the natural microbial community in order to accomplish an effective bioremediation technique in the field. Substantial laboratory- and mesocosm-based research is essential to assess the potential of bioremediation, although it must be recognised that environmental factors will play an important role in determining the actual degradation rate in the field. While in controlled laboratory trials, measurements can generally be interpreted easily; cause-and-effect relationships are often hard to establish at field sites. In most bioremediation cases microorganisms can readily degrade the contaminant when grown in well-controlled laboratory environments; however, evidence of field biodegradation is necessary. When degrading microbes are introduced into less hospitable environmental conditions in the field, they may not perform the same tasks and can be inhibited because of predation or competition by autochthonous microorganisms [86]. In addition, because of the heterogeneity of oil, evaluation of petroleum hydrocarbon degradation at the field scale is more difficult. The bioremediation process can be influenced by both biotic and abiotic factors as well as the ability of microorganisms to survive and migrate. Therefore, it is necessary to conduct laboratory experiments prior to the actual cleanup process to assess the improvement of hydrocarbon degradation under controlled conditions; this will establish the scientific credibility of a specific bioremediation procedure.

## 8. Factors Influencing Biodegradation of Hydrocarbons

The key purpose of remediating polluted sites is to diminish the hazard of contaminants to human health as well as the environment through the application of optimal remediation techniques [22]. As already discussed the application of bioremediation technologies in the actual environment (field) is challenging as hydrocarbon biodegradation in soil is determined by a number of environmental and biological factors varying from site to site [87]. Parameters influencing bioremediation include the nature and concentration of the contaminants, type and structure of the soil and the presence and survival of contaminant-degrading microorganisms. Environmental conditions such as the pH of the soil, oxygen availability and nutrient content can also limit the bioremediation process by inhibiting the growth of hydrocarbon-degrading microbes and/or reducing the bioavailability of pollutants to microbial attack.

Limited bioavailability of hydrocarbons to microorganisms can result in a less effective bioremediation process by limiting the rate of hydrocarbon degradation. The interaction between hydrocarbon degrading microbes, soil matrix and the contaminants plays an important role in the bioremediation process. Soil organic matter is one of the most significant factors having a dominating influence on the interactions between soil and the organic pollutant [88]. The percentage of soil organic matter controls the partitioning of petroleum hydrocarbons into the organic fraction of soil and the extent of sorption, affecting the degradation rate. The impact of soil organic matter on the degradation of petroleum hydrocarbons has been clearly shown in many studies [89].

The degradation rate of petroleum pollutants is generally higher during the early stage when the pollutants are easily bioavailable; in contrast in the second remediation stage contaminant bioavailability is limited as a result of hydrocarbons being sequestered. Generally once this stage has been reached no further degradation occurs during the rest of remediation [90]. A high concnetration of organic matter in the soil results in the organic matter acting as a partitioning medium, resulting in the sequestration of contaminants which partition into the organic fraction resulting in reduced degradability of contaminants [91].

Like soil organic matter, ageing of the polluted soil can also adversely affect the degradation of petroleum hydrocarbons. A number of studies have reported greater rates of hydrocarbon degradation in freshly polluted soils compared with aged hydrocarbon fractions. This may result from the contaminant being partitioned into the soil organic matter fraction or penetration into small pores leading to a decline in pollutant bioavailability to microbes [91]. This problem is more obvious in soils with high levels of organic matter than in that with low organic matter [92]. Hydrocarbon properties are also different in fresh petroleum products from that found in aged products. Hydrocarbon aging thus results in a reduced rate of degradation in the early stages.

The main factors and their impact on the feasibility and rate of petrogenic hydrocarbon biodegradation in soil are summarised in Table 4.

**Table 4.** Factors and their effect on the degradation of petroleum hydrocarbons in the polluted soil.

Factor	Description and effect on bioremediation rate	Reference
**Temperature**	Temperature affects rates of hydrocarbon degradation and the physico-chemical composition of oil, result in enhanced hydrocarbon bioavailability as well as the composition and metabolic activity of the microbial communities. In soils 30–40°C is the temperature range in which the highest degradation rates generally occurs. Increased temperature also decreases oil viscosity, increases hydrocarbon solubility, hastening the diffusion of hydrophobic pollutants and enhancing degradation rates of hydrocarbons.	[93]–[96].
**Nutrients**	The absence of or low levels of key nutrients in the soil directly affects microbial cell growth and activity. Optimal level of nutrients is essential for higher hydrocarbon-utilising microbial activity. Excessive amounts of nutrients such as NPK in the soil can also negatively affect the biodegradation of hydrocarbons resulting in inhibition of the microbial biodegradation activity.	[78],[93]
**Characteristics and concentration of petroleum hydrocarbons**	The rate at which hydrocarbon-utilising microorganisms breakdown the hydrocarbons depends upon hydrocarbon characteristics including chemical structure and concentration of these pollutants. Petroleum fractions, n-alkanes of intermediate length (C_10_–C_25_) are preferred and more degradable. Longer chain alkanes (C_25_–C_40_) are hard to degrade due to their hydrophobicity, poor water solubility and bioavailability. Branched chain alkanes and cycloalkanes degrade more slowly than the corresponding unbranched alkanes. Complex and less soluble compounds result in reduced hydrocarbon degradation rates. High concentrations of hydrocarbons are toxic to microorganisms involved in hydrocarbon degradation, as they affect their growth and activity.	[2]
**Bioavailability**	The rate of degradation determined by the bioavailability of hydrocarbons. As the molecular weight of hydrocarbons increases, the solubility of these pollutants decrease resulting in lower accessibility of hydrocarbons for metabolism by the microbial cell. PAHs are hydrophobic compounds with low bioavailability and rapid sorption to organic matter and soil matrix making them recalcitrant. The longer the contact between soil and hydrocarbon contaminants the more irreversible the sorption, and the lower is the extractability of the pollutants from the soil.	[2]
**Soil Characteristics**	The structure and conditions of the soil determine the movement of the pollutants, thereby affecting the rate of biodegradation. High concentrations of soil organic matter in fine soil enhances bacterial growth and stimulates the biodegradation of hydrocarbons. A higher rate of degradation of hydrocarbons occurs in silty soil compared to sandy soil due to the poor microbial content in the sand fraction which corresponds to a high C: N ratio and lower internal surface structure.	[97],[98],[99]
**Oxygen availability and transport**	Dissolved molecular oxygen soil and the requirements for its delivery are crucial keys for the success of the bioremediation process. The importance of oxygen derives from the respiration process and the participation of oxygenases in the subsequent degradation pathway of the hydrocarbons. For example, in soil it usually takes 2 × 10^6^ m^3^ of water saturated at 10 mg/litre O_2_ to effectively oxidize 10 m^3^ of hydrocarbon to carbon dioxide and water. Oxygen availability in soil is reliant on soil type, moisture content and the rate of biodegradation.	[2],[100]
**Microbial presence of active hydrocarbon-utilising microorganisms**	Microbial strains which have the ability to survive in the presence of pollutants and use them as a source for growth and metabolism are the dominant microorganisms in the contaminated soil. The number of hydrocarbon degrading organisms in the contaminated soil determines the rate of degradation; a lack of these microbes leads to a reduced hydrocarbon degradation rate. The contaminated soil must contain a sufficient number of hydrocarbon-utilising microorganisms, specifically those which are active. As a result of bioremediation, the active microbes may increase the microbial community in the soil. A lack of hydrocarbonoclastic microbes in the contaminated soil can be overcome by inoculating the soil with a selection of appropriate strains to biodegrade contaminants (bioaugmentation).	[22],[101]
**Eco-toxicity**	Petroleum hydrocarbons have a toxic effect on bacterial activity, some plant species and earthworms resulting in reduced biodegradation rates.	[102],[103]

## 9. Conclusion

The widespread utilisation of petroleum hydrocarbons in different industrial applications presents a challenge for the remediation of polluted sites. In most cases, the ability of these contaminants to sorb onto mineral and organic matter of the soil determines the efficiency of the remediation process. A significant amount of bench-scale work has concentrated on the ability of a diversity of microbes including bacteria and fungi to transform these complex compounds. The pathways of aerobic and anaerobic degradation of petrogenic hydrocarbons have been established and this has led to an interest in the potential use of microbes to degrade petroleum-contaminated sites. To date, bioremediation approaches have shown significant promise. However, further research to overcome several implementation issues is required. In addition, more field and pilot scale trials are important to evaluate the efficiency of these processes, taking into consideration that each site is different and numerous factors must be considered. An effective remediation of a contaminated site depends on the appropriate selection and design of the remediation technique.
